# Fluid leakage near the percolation threshold

**DOI:** 10.1038/srep19513

**Published:** 2016-02-03

**Authors:** Wolf B. Dapp, Martin H. Müser

**Affiliations:** 1 Forschungszentrum Jülich, John von Neumann Institut für Computing and Jülich Supercomputing Centre, 52425 Jülich, Germany; 2 Universität des Saarlandes, Lehrstuhl für Materialsimulation, Saarbrücken, Germany

## Abstract

Percolation is a concept widely used in many fields of research and refers to the propagation of substances through porous media (e.g., coffee filtering), or the behaviour of complex networks (e.g., spreading of diseases). Percolation theory asserts that most percolative processes are universal, that is, the emergent powerlaws only depend on the general, statistical features of the macroscopic system, but not on specific details of the random realisation. In contrast, our computer simulations of the leakage through a seal—applying common assumptions of elasticity, contact mechanics, and fluid dynamics—show that the critical behaviour (how the flow ceases near the sealing point) solely depends on the microscopic details of the last constriction. It appears fundamentally impossible to accurately predict from statistical properties of the surfaces alone how strongly we have to tighten a water tap to make it stop dripping and also how it starts dripping once we loosen it again.

Seals and gaskets are crucial components in many hydraulic systems such as water taps, pipes, pumps, or valves^1^. Their main function is to prevent undesired or uncontrolled leakage of gases or fluids from one region to another. Despite their importance, attempts to estimate leak rates of seal systems from first principles succeeded for the first time only less than a decade ago^2^,^3^,^4^. Earlier treatments could not accurately predict the distribution of microscopic interfacial separations in a mechanical contact, which is needed for the fluid-mechanics aspect of the problem. Persson’s contact mechanics theory^5^,^6^ provides this information and computes the leakage in terms of Bruggeman’s effective-medium approximation^7^ to Reynolds’ equation^8^.

In a previous publication^9^, we demonstrated that Persson’s contact mechanics theory, combined with a slightly modified version of Bruggeman’s effective-medium approximation, reproduced almost perfectly the results of computer simulations, in which an ideally well-defined leakage problem was solved to high numerical precision. The favourable comparison of theory and simulations benefited to some degree from fortuitous error cancellation: Persson theory slightly underestimates the rate at which (mean) gaps diminish with increasing load, which almost exactly compensates the minor overestimation of leakage in Bruggeman’s approximation. Persson’s treatment is therefore certainly accurate enough to explain why leakage through interfaces decreases roughly exponentially with the mechanical load^3^,^4^,^10^ pressing two (elastic) bodies against each other, where at least one of them has a self-affine rough surface (see the method section).

Although Persson theory has proven successful in describing leakage over a broad parameter range, one cannot expect it to hold near the sealing transition. One reason is that mean-field theories like Bruggeman’s, which is part of Persson’s approach to leakage, are known to fail near critical points, even if they perform quite well outside the critical region^11^. Alternative, percolation-theory-based treatments of leakage^12^,^13^ or related approaches assuming that most of the fluid pressure drops near a single, narrow constriction (or a two-dimensional network of constrictions)^3^ also risk to fail in the vicinity of the sealing transition. This is because length and width of an isolated constriction show different scaling with the applied load^14^ in contrast to assumptions made in the respective theories.

In this work, we investigate the fluid leakage through a mechano-hydraulic interface by means of computer simulations. In contrast to previous studies^9^,^15^,^16^ our focus lies on calculating leakage between randomly rough bodies near the percolation threshold. A particular motivation to revisit the problem stems from our observation that local details, such as the presence or absence of adhesion between the surfaces, affect the conductance exponent of isolated constrictions^14^. It remains unclear if or to what degree the critical behaviour (evaluated near but not too close to the percolation threshold) is determined by the disorder at large length scales, which is usually considered central in percolation theory^17^.

## Results and Discussion

### Adhesion-free sealing transition in the continuum limit

We begin the analysis of leakage near the percolation threshold by simulating our “default model”. It is based on approximations that are commonly made to study either the contact-mechanics or the fluid-mechanics aspects of our leakage problem: self-similar surface roughness, linearly elastic bodies, small surface slopes, and absence of adhesion between the surfaces. Fluid flow through the interface is treated in terms of the Reynolds equation. Some of the approximations of our default model are relaxed below. More details are given in the method section.

**Figure 1:**
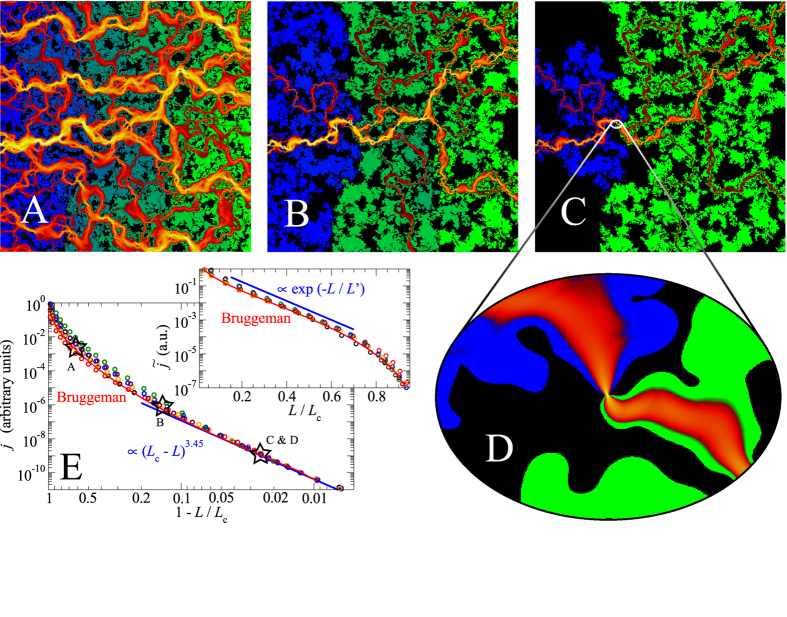
(**A**–**D**) Visualisation of the fluid flow through a microscopically rough contact at different loads, as indicated in panel E. Black colour marks regions that do not belong to the percolating fluid channel. Blue and green colours indicate the fluid pressure, which drops from one (blue) on the left-hand side of the interface to zero (green) on the right-hand side. Red and yellow indicate the absolute value of the fluid current density. (**E**) main panel: Double-logarithmic representation of the mean leakage current *j* as a function of the reduced load 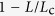. Differently coloured symbols represent different random realisations of the surface roughness. Data is shifted vertically (by as much as a factor of 10) to superimpose in the critical region. In the inset of panel (**E**), the dimensionless load 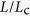 is plotted linearly and the current is now normalised (shifting factors 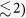 such that it superimposes in the domain where it decreases exponentially with load. Red lines show the predictions of effective medium theory, modified such that it reproduces the exact critical load for a given random realisation (see ref. 9).

Leakage flow for our default system is shown in Fig. 1E for different reduced loads 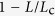, where *L* is the absolute load squeezing the surfaces together and 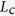 is the critical load, defined as the largest load at which at least one fluid channel still percolates from the right to the left side of the interface. Our data is based on different surfaces, which are produced with identical stochastic rules but different random seeds. To enhance sampling, we also considered inverted and 90° rotated surfaces. All realisations show similar behaviour: For very small loads, the current decreases very quickly with increasing load before the dependence becomes roughly exponential. At 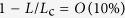, a crossover to a powerlaw ensues


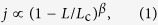


where *β* is the conductance exponent. The value of *β* deduced from the data is consistent with the one we identified for isolated, single-wavelength constrictions^14^, i.e., 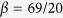. This value is much greater than typical conductance exponents for seemingly related percolation problems such as the two-dimensional random (on/off) resistance network, for which 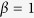
18. Surprisingly, Bruggeman’s effective medium approach predicts the current quite accurately for most random surface realisations investigated in this study, even close to the percolation point and in all cases does it find cross-over loads within roughly 10% percent at which the exponential load-current relation ceases to be valid.

To rationalise how the mean fluid flow develops as a function of load, it is instructive to visualise the spatially resolved fluid pressure and current density for a particular random realisation. This is done in Fig. 1A–D. In the domain where flow decreases exponentially with load, the fluid pressure drops in a quasi-discrete fashion at a number of constrictions. These constrictions are distributed seemingly randomly throughout the interface thereby roughly mimicking the conditions assumed in the derivation of Bruggeman’s effective-medium theory. Once the fluid pressure drops predominantly at a single constriction, see Fig. 1C,D, mean-field theory may still be correct, albeit only incidentally. In the language of percolation theory, all current now goes through one hot bond. In contrast to assumptions commonly made for random disorder, the resistivity assigned to individual points is not discrete but it changes continuously with the control parameter and eventually diverges at the critical point. In our case, the control parameter is the load, while in most percolation models it would be the probability with which a bond (or a vertex point or an individual point in a continuous domain) would be assigned a (fixed) finite or infinite resistance.

Our system can be characterised as having correlated disorder^19^ (if the gap is large at a given position, then the gap is likely also large nearby) and at the same time long-range interactions^20^ (the elastic Green’s functions in real space decay with 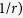. Apparently, the way in which these two ingredients are combined here turns percolation of seals into a local problem such that it is not possible to assign a (unique) universality class to the leakage problem, even if the stochastic properties of the problem are fully defined. We note that neither changing the Hurst exponent nor increasing system size alters the observed behaviour in a qualitative fashion. While increasing the range over which the surface spectrum is self similar can and does affect the low-load flow quite dramatically, the critical region does not appear to be affected, at least not for practically relevant spectra, in which self-similarity is rarely observed for more than five or six decades in wavelength. In all cases we find that critical behaviour, i.e., the range of loads in which equation (1) holds to within a few ten percents, starts to set in at roughly 0.8 to 0.9 times the critical load. We substantiated these claims by running additional simulations for 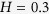, by extending the ratio of roll-off wavelength 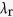 and short wavelength cutoff 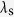 from 64 to 256 (and within Persson theory to 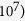, and by extending the ratio of system size 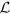 and 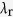 from 2 to 16.

### Size-dependence of the critical regime

In the critical regime, the pressure drops predominantly at a single constriction. One might argue that large systems have a smaller critical regime, because significant pressure drops can then occur at several constrictions. We therefore analysed how the size of the critical regime depends on the system size. For this, we changed the ratios 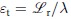 and 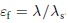. Here, we may associate 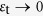 with the thermodynamic limit and 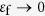 with the fractal limit. In real applications, the true (mathematical) limits have no significance, which is why we content ourselves with projections of our results to more realistic values of 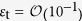 and 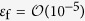.

**Figure 2:**
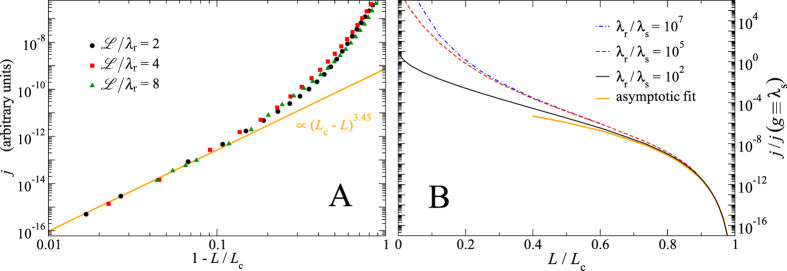
Size-dependence of the critical regime. Panel (**A**) Fluid current *j* as a function of reduced load for different ratios of system size and rolloff wavelength 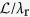. The current is shifted vertically to superimpose the data close to the percolation threshold. Other dimensionless quotients are kept constant, e.g., 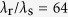 and 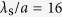. Panel (**B**) Fluid current *j* in Persson theory as a function of the normalised load 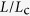 for different ratios of roll-off wavelength and short-wavelength cutoffs, 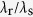. As the normalising factor for *j*, we chose the current that one would obtain if the gap *g* were set to 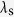 everywhere.

Figure 2A reveals that changing 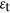 does not have a sufficiently strong systematic effect to dominate the fluctuations between different random realisations, at least not when changing 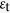 by a factor of four, i.e., for our three choices of 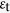 there is not even a monotonic trend. We note that we carried out a disorder average for 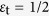 over 16 different realisations but considered the large system 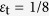 sufficiently self-averaging. Since 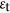 is never a very small number in practice, we conclude that the critical leakage regime in real applications should not be much reduced in size compared to the calculations presented here.

We also changed 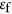 and trends on the size of the critical regime were again difficult to ascertain, due to large statistical scatter. We therefore considered realistic values for 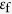 within Persson theory, where we proceed for the calculation of the gap distribution function as in ref. 21, and present our results on the mean flow for 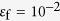 and 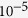 as well as a rather small value (irrelevant for practical applications) of 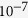, see Fig. 2B. The results for the analysed values of 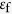 are very close at loads approaching the critical load. While the critical region is slightly reduced for small values of 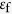, it is clearly revealed that decreasing 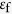 below 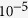 has only marginal effects at loads exceeding 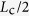. This could have been expected from the following argument: decreasing 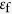 corresponds to adding roughness at long wavelengths. Since the effective elastic compliance decreases with the inverse wavelength, this extra roughness is immediately accommodated by the elastic seal. We conclude that from a mathematical point of view the expected flow or conductivity has a fractal limit, which is only (approximately) reached in practice for loads not too small compared to the critical load.

### Critical leakage for negative slip lengths

Conductance exponents in percolation theory frequently turn out to be universal, that is, they remain unaltered when details of a model change. In view of this finding, we explore whether the conductance exponent also remains unaltered with small alterations to the default leakage model. One simple modification is to assume that the fluid flow velocity does not extrapolate to zero precisely at the walls (so-called stick condition) but already a distance 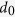 before the wall. In fluid dynamics, 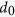 is called a (negative) slip length. In a recent work^22^, softening the fluid-obstacle repulsion, in effect using a positive sliplength, suppressed the expected, universal critical behaviour for 2D particle transport through porous media, albeit for non-correlated obstacles.

In the present context, one could argue that a negative slip length accounts for the finite size of particles to lowest order: the fluid particles can only penetrate gaps with a height greater than 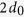. The local fluid conductivity now scales with 
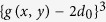
 rather than with 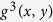, where 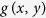 is the gap as deduced from the contact-mechanics calculation at an in-plane coordinate 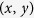. To analyse the effect of finite, negative slip lengths, we solve the Reynolds equation for the same gap topography as before, but using the just-described conductivity. We choose 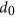 to be a small fraction of the root-mean-square height 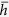 of the rough substrate. We varied this fraction by a factor of 1000 without a qualitative change of the observations.

**Figure 3:**
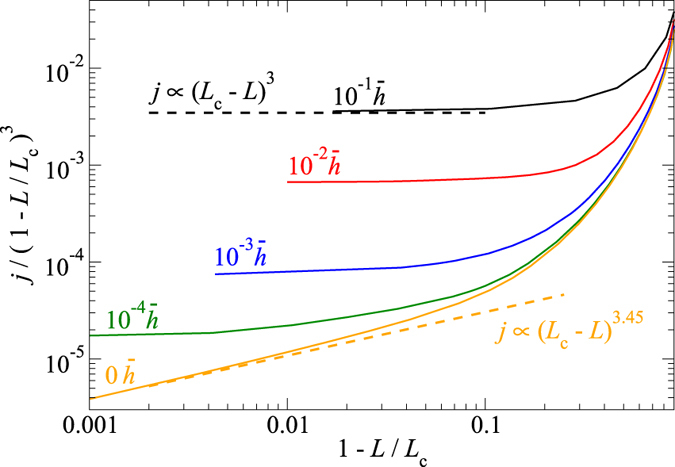
Leakage current *j* as a function of the reduced load 1–*L/L*_c_ for a system with a negative slip length for the fluid flow. The same random realisation is studied as in Fig. 1A–D, where zero slip-length flow is considered. The data is smoothed to remove scatter and shifted vertically to yield a current of one at an infinitesimally small load.

Figure 3 reveals that the flow is not affected far from the percolation point. However, the conductance exponent now appears to be 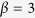. This value can be readily explained: in the present model, the constriction (which is located around the point where the substrate height has a saddle point) is not yet fully closed when it appears closed for the fluid. Thus, the point is only critical for the flow but not for the contact mechanics. This means that near the critical load 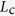, the true height of the gap at the saddle point, 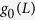, the true length of the constriction 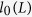, and the true width of the constriction 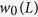 are all “simple functions” of the load, which each can be expanded into a Taylor series according to 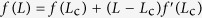. The same quantities, as perceived by the fluid, e.g., the effective local height, or the effective width of a constriction, have similar functional dependencies as the true height, however, different offsets. In fact, all offsets 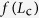 for the effective quantities can be set to zero, since height, width, and length of the constriction—“as seen by the fluid”—are all zero at the critical point. Since the resistivity of the constriction scales as the inverse third power of the effective gap, linear with the length of the constriction (as in a serial coupling of resistors) and with the inverse width of the constriction (as in a parallel coupling of resistors), the fluid resistance of the constriction follows


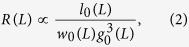


where the proportionality factor is linear in the viscosity of the fluid and also depends on the geometry of the constriction. Inserting our Taylor series approximations for effective height, width, and length of the constriction into Eq. (2) then reveals that 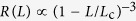 implying 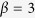. As discussed in a previous paper^14^, the case of zero slip length is more complicated, because 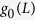, 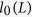, and 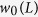 all approach zero as non-integer powerlaws of the reduced load.

### Critical flow through adhesive interfaces

We now turn our attention to surfaces that attract each other via adhesive forces. The fluid flow has stick boundary conditions again. In a previous work^14^, we found, for the case of isolated constrictions and short-range adhesion, that constrictions closed discontinuously. Long-range adhesion was not considered explicitly as it reduces to a simple adhesive offset force for the investigated single-wavelength isolated constriction (see the method section). The range of adhesion is quantified by a dimensionless number called the Tabor coefficient 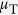
23. Its use is best known in the context of single-asperity contacts, but the concept extends to randomly-rough, self-affine surfaces^24^. Except for prefactors, which can be chosen at will^25^, it is defined as 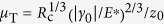. Here, 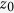 is a characteristic length scale of the interaction, 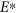 is the effective elastic contact modulus, and 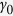 is the surface energy. 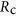 is the radius of curvature for a Hertzian contact geometry, or a measure for the inverse surface curvature: 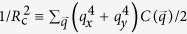 where 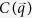 is the (discrete) wavevector-dependent height spectrum defined in the method section.

**Figure 4:**
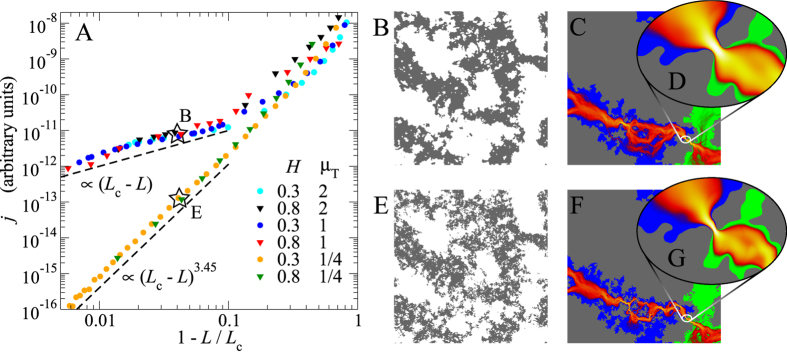
(**A**) Leakage current *j* as a function of the reduced load 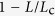 for adhesive contacts differing in their Hurst roughness exponent *H* and Tabor coefficient 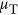. Data is shifted vertically to superimpose in the critical regions. (**B**,**E**) show the true contact near the percolation threshold for one random realisation with 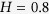, in the case of short-range 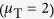 and long-range 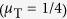 adhesion, respectively. (**C**,**F**) are the corresponding flow patterns at loads indicated in panel (**A**). Panels (**D**,**G**) are high-resolution zooms into the critical constriction.

Figure 4 shows that the critical leakage current sensitively depends on the adhesive range. Long-range adhesion yields a similar dependence of the current on the reduced load as the non-adhesive case. However, at a Tabor coefficient around 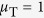, the leakage-load dependence starts to show a different powerlaw near the sealing transition. Specifically, for 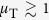, load regimes with an apparent conductance exponent of 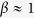 occur. We thus have the second example for a change of conductance exponent of macroscopic, or at least mesoscopic, response functions due to small changes in the model. Below, we demonstrate that the observed crossover is also present in an isolated constriction and thus not due to the multi-scale topology of the percolating channel.

An interesting aspect of Fig. 4 is that the coarse features of the contact area look almost identical near the sealing transition, even in the two extremes of no adhesion and short-range adhesion. However, the contact lines look much smoother and less fractal for short-range adhesion (E) than for no or long-range adhesion (B). This difference in local contact features ultimately accounts for the different behaviour near the percolation threshold.

### Flow through isolated, adhesive constrictions

**Figure 5:**
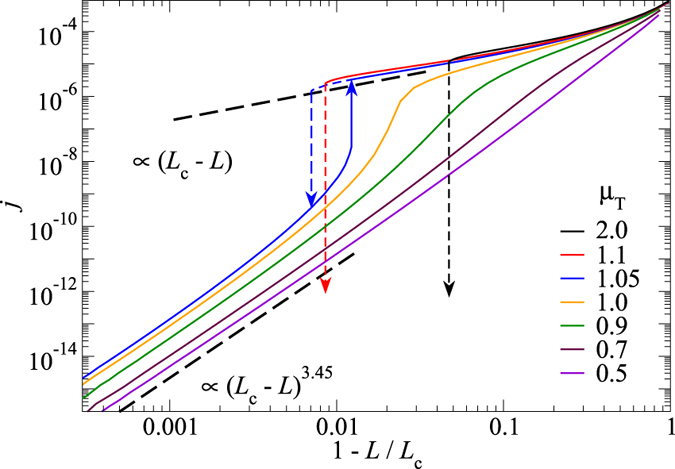
Leakage current *j* as a function of the reduced load 1–*L/L*_c_ for an isolated, single-wavelength constriction, for different Tabor parameters.

We now address the question of whether the crossover of exponents presented in Fig. 4 is due to the multi-scale roughness of the surfaces or originates from the properties of an isolated constriction. Towards this end, we revisit the contact mechanics of single-wavelength roughness, in particular that of a square saddle point (for details see ref. 14). However, here we do not only consider short-range adhesion as in our precedent study on isolated constrictions, but also allow for medium- or long-range adhesion. Figure 5 shows that for 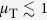 the scaling of the current on the load changes near the percolation point, from the 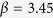 behaviour also seen in the non-adhesive case, towards a scaling with 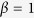. For 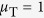, the latter regime is rather narrow and the leakage quickly becomes similar to that of non-adhesive surfaces as the sealing transition is approached. In a narrow range of 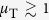, the “new” scaling is valid over more than one decade. For 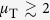, there seems to be a discontinuous drop of finite to zero conductance of the critical junction. The critical constriction snaps shut before scaling can be observed. With decreasing range of the adhesive potential, this point of adhesive instability is moved to smaller loads, and away from the critical load.

## Conclusions

From the three leakage models analysed in this study, it has become clear that seal systems are unlikely to belong to a (unique) universality class of percolation theory, even though one might consider flow through a seal as a paradigm percolation problem. When adding small alterations to our default model, in which common approximations of lubrication theory are made, we observe qualitative changes in the transition between finite and zero conductance. In fact, it appears as though the default model represents a multi-critical point, because the conductance exponent changes when an arbitrarily-small negative slip length is introduced and/or the transition changes from continuous to discontinuous when short-range adhesion between the surfaces is introduced. In practice, many additional complications can and in general will affect the leakage problem, most notably the formation of fluid capillaries, clogging by contaminating particles, as well as viscoelastic deformation and ageing of the sealing material. These complications are likely to correspond to relevant perturbations affecting the nature of the percolation transition as well.

While it is certainly possible to predict leakage over a broad pressure range from statistical properties of the surfaces alone, it appears impossible to do so accurately when the load exceeds 80% or 90% of the critical load, above which no open channel percolates from one side of the interface to the other side. At such high loads, most of the pressure pushing the fluid through the interface drops at a single constriction. The (local) properties of this constriction then determine the behaviour of the whole system. This is one reason why it is impossible to predict with great accuracy how strongly we have to tighten a water tap to make it stop dripping and also how it starts dripping once we loosen it again.

## Methods

### GFMD

The contact mechanics treatment and its description is in large parts identical to that presented in our study on isolated constrictions^14^: We assume linear elasticity and the small-slope approximation, so that the roughness can be mapped to a rigid substrate and the elastic compliance to a flat counter body without loss of generality. The effective contact modulus is used to define the unit of pressure, i.e., 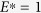. Elasticity is treated with Green’s function molecular dynamics (GFMD)^26^ and the continuum version of the stress-displacement relation in Fourier space, 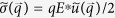, where 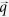 is a wave vector and *q* its magnitude. Simulations are run in a force-controlled fashion. After the external load has changed by a small amount, all degrees of freedom are relaxed until convergence is attained.

Two surfaces interact with a hard-wall constraint, i.e., they are not allowed to overlap. In addition, we assume a finite-range surface energy 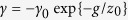, where 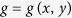 is the local gap or interfacial separation. When adhesion is switched on, we choose 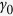 such that the contact area roughly doubles at a given load compared to the adhesion-free case.

The linear size of the solids is denoted by 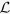. Periodic boundary conditions are employed within the *xy*-plane so that the local height in real space can be written as a Fourier sum 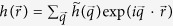.

Most technical and natural surfaces are self-affine rough, e.g., ground steel, asphalt, human skin, and sandblasted plexiglass show self-similar height spectra over a broad range with Hurst roughness exponent of *H* ≈ 0.8^27^. The exponent states that the root-mean-square deviation of the height increases as 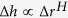, where 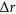 is the in-plane distance from a given point on the surface. An ideal random walk corresponds to 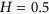. A larger value of *H* indicates that height spectra increase in magnitude at long wavelengths relative to short wavelengths. Sometimes 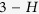 is called the fractal dimension of the surface. Pertaining to the single-wavelength *λ* constrictions discussed in ref. 14 and in the appendix, we note that their equilibrium height-profile can be generated, for example, from 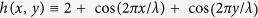.

The roughness spectra 
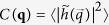
 is constant for wave vectors of magnitude 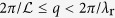, where 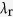 is called the rolloff wavelength. For wave vectors of magnitude 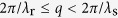, the spectra are power laws according to 
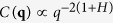
. A typical setup can be characterised by the following dimensionless numbers: 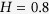, 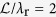, 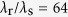, and 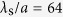. When approaching the percolation threshold or when treating short-range adhesion, we further increase the ratio of 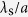 while keeping the random realisation of the surface profile the same. To determine fluctuations of the fluid conductance at small loads, we decrease 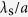 but take much larger ratios for 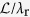 and 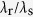. The largest GFMD calculation presented in this work consisted of 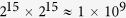 discretisation points.

### Reynolds solver

For the fluid-mechanics-related calculations, we assume a reservoir of liquid on the left side of the system 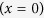, while the other, right side is a sink for said liquid, with a liquid pressure of zero. In the transverse direction, the system is treated as periodic, in order to minimise finite-size effects. The local fluid conductivity in the Reynolds equation scales with the third power of the local gap as seen by the fluid.

Like the contact-mechanics aspects, all solution strategies including their descriptions are in large parts identical to those presented in our study on isolated constrictions^14^: We use the *hypre* package^28^ to solve the sparse linear system that the discretised Reynolds equation can be expressed as. We employ the solvers supplied with *hypre* using the CG (conjugate gradient), or GMRES (generalised minimal residual) methods^29^, each preconditioned using the PFMG method, which is a parallel semicoarsening multigrid solver^30^. Our in-house code is MPI-parallelised and uses HDF5 for I/O.

The fluid pressure and its gradients are assumed small enough to not deform the walls. We verified that our results for the conductance exponent do not depend on this approximation, by including the coupling of the fluid pressure (up to 30% of the external mechanical pressure near the percolation threshold) to the wall for an isolated (adhesionless, zero-slip length) constriction. We merely observed an increase in the percolation load, a shift in the location of the critical constriction, and a reduction of symmetry of the gap and contact line profiles. Coupling to GFMD is done through an iterative perturbation treatment, in which the Reynolds output is fed back into the contact mechanics calculation.

## Additional Information

**How to cite this article**: Dapp, W. B. and Müser, M. H. Fluid leakage near the percolation threshold. *Sci. Rep.* **6**, 19513; doi: 10.1038/srep19513 (2016).
